# Extraordinary Case of Mortification, Occasioned by Pregnancy

**Published:** 1853-04

**Authors:** Samuel L. Hamilton

**Affiliations:** Chattooga county, Georgia


### OBSTETRICS.

*Extraordinary Case of Mortification, occasioned by Pregnancy.* By Samuel L. Hamilton, M. D., of Chattooga county, Georgia.—Mrs. S., aged twenty years, during the sixth month of her second pregnancy, was attacked June 5th, 1848, with remittent fever, which readily yielded to ordinary treatment. After this she did well for twelve or fifteen days, during which time nothing of importance occurred, but she was perhaps unusually hearty, which I attributed to her condition.

On the morning of the 20th of June I was again called to see her, and found her with extreme pain in the toes of her right limb, which was so cold half way to the knee as to yield to the hand when applied the same sensation as that of a dead body. There was little or no excitement of the pulse; nor was there any evidence of disease in the general system. The pain in the foot increased from day to day, until the cries and shrieks of the unfortunate sufferer were almost too intolerable to be borne by her surrounding friends. It cannot be said that she was entirely easy until her delivery, except while under the influence of large doses of morphine, from the effects of which she would frequently awake in violent pain. Her sleep was evidently not profound, under as large doses of the above medicine as it is thought prudent to administer under any circumstances. It was attempted, but in vain, to restore the lost temperature of the extremity—every thing was used that would promise this result, without vesicating the skin, which was avoided, notwithstanding the repeated solicitation of friends for the application of a fly-blister. There finally appeared large yellow blisters upon the diseased surface, which, on being ruptured, discharged a serous fluid. It was evident, after this, that there was not sufficient vitality in the skin to warrant recovery. The skin and all the soft tissues now began to slough, until the ligaments of the metatarsal articulations were destroyed—at this point they were separated, which gave the patient some slight relief. The temperature of the limb was partially restored from above, down to within a short distance of the ankle-joint; the pain, however, continued unabated till labor came on, when it then entirely ceased. The lady was then delivered of a large child, after which there was not the slightest return of pain. The labor was in all respects natural—the stump of the extremity, which had been suppurating, now readily healed—the woman recovered, and has since given birth to two children, without the slightest return of the affection.

I was constrained to the opinion that this extraordinary case was the result of pressure of the gravid uterus upon the arteries and nerves passing through the pelvis—viz., the ext. iliac artery and the great sciatic nerve, whose extremities supply nutrition and innervation to the disorganized part. From the history of her previous pregnancy, it appeared that there had been running ulcers from about the sixth month until delivery; the cicatrices were still perceptible, and occupied the regions covering the posterior and anterior tibial arteries. I think it a reasonable inference, that the pressure was exerted upon the nerves, as well as the arteries, from the intense and continuous pain which nothing could relieve until the removal of the cause, when the suffering simultaneously disappeared.

As to the treatment of the case, opium may have given some temporary relief. Position was tried without the least success. Supporters were also tried with the same result. We would feel inclined, however, were we called to treat another case, to try more thoroughly both supporters and position. We might particularize as to local applications, &c., but they were such as common sense would dictate, and failed to afford any relief—we therefore omit their tedious detail.—*Southern Med. and Surg. Journ.*

